# HLA-B*1502 in Iranian Children with Anticonvulsant Drugs-Induced Skin Reactions

**Published:** 2017

**Authors:** Seyed Hasan TONEKABONI, Narjes JAFARI, Mahboubeh MANSOURI, Sayena JABBEHDARI, Rahil EFTEKHARI, Zahra CHAVOSHZADEH, Fatemeh ABDOLLAH GORJI, Mehrnaz MESDAGHI

**Affiliations:** 1 Pediatric Neurology Research Center, Shahid Beheshti University of Medical Sciences, Tehran, Iran; 2 Pediatric Neurology Department, Mofid Children’s Hospital, Shahid Beheshti University of Medical Sciences, Tehran, Iran; 3 Department of Immunology and Allergy, Mofid Children’s Hospital, Shahid Beheshti University of Medical Sciences, Tehran, Iran; 4 Department of Immunology, School of Medicine, Tehran University of Medical Sciences, Tehran, Iran; 5 Clinical Research Development Center, Mofid Children’s Hospital, Shahid Beheshti University of Medical Sciences, Tehran, Iran

**Keywords:** Drug eruption, HLA- B*1502, Anticonvulsants

## Abstract

**Objective:**

Anticonvulsant drugs can cause various forms of skin drug reactions, ranging from exanthema to severe blistering reactions. An association between HLA-B*1502 allele and severe skin reactions have been reported.

**Materials & Methods:**

Fifteen patients with severe skin reactions following treatment with anticonvulsant drugs (Carbamazepine, lamotrigine, phenobarbital, primidone) and 15 controls (age-matched epileptic patients taking similar anticonvulsants without drug eruption) were included. They were referred to Mofid Children’s Hospital in Tehran, Iran, between Jan 2012 to Jan 2014. Genomic DNA was extracted from peripheral blood of all patients and HLA- B*1502 genotype was detected by real-time PCR.

**Results:**

None of the patients was positive for HLA- B*1502, but two patients in control group had positive HLA- B*1502.

**Conclusion:**

The HLA- B*1502 is not correlated with severe anticonvulsant drugs -induced skin reactions in Iranian children.

## Introduction

Epilepsy is one of the most common serious neurological disorders and the cornerstone of its treatment is usage of anti-epileptic drugs. A minority of patients under treatment with anticonvulsant drugs shows broad-spectrum skin reactions as an adverse effect. These reactions include Stevens–Johnson syndrome (SJS), toxic epidermal necrolysis (TEN) and drug reaction with eosinophilia and systemic symptoms (DRESS), classified according to skin manifestations and percentage of body surface involved) 1, 2). Genomewide approach has been used to identify HLA alleles and genetic predisposing factors for drug-induced skin reactions (3).

In some Asian and European countries, HLA- B*1502 and HLA-A*3101 has been identified as predictive genetic markers for carbamazepine hypersensitivity (4).

Recent studies have shown an association between HLA-B*1502 and antiepileptic drug-induced cutaneous reactions, including carbamazepine, phenytoin, and lamotrigine (5, 6).

These reactions have high morbidity and can be lethal. Screening of children, who are genetically vulnerable to these skin reactions, can help us to prevent these complications. As there is no data about the relationship of HLA- B*1502 and severe drug reactions to anti-epileptic drugs in Iranian children, we designed this study to elucidate the importance of HLA- B*1502 in adverse reactions.

## Materials & Methods

This cross-sectional descriptive study was performed on 15 patients, referred to Mofid Children’s Hospital in Tehran, Iran, between Jan 2012 to Jan 2014, with severe skin reactions (SJS,TEN, overlap syndrome and DRESS) after anticonvulsant drug consumption (Carbamazepine, lamotrigine, phenobarbital, primidone) and compared to 15 patients with seizure disorders, treated with similar anticonvulsant drugs for at least 3 months and had not shown any adverse reaction (control group). The case and control groups were age and sex matched. 

The patients’ data were recorded in a questionnaire including age, gender, type of reaction, type of the anti-epileptic drug used, dosage and duration of drug consumption, history of gastrointestinal and respiratory hypersensitivities, concomitant symptoms such as fever and lymphadenopathy, type of seizure including EEG findings, Brain MRI and laboratory findings (CBC, diff, eosinophilia, ESR, AST, ALT). A clinical immunologistallergist, based on skin reaction criteria (7), confirmed diagnosis of the type of skin reaction. 

After signing an informed consent by the patients’ parents, 3 mL EDTA blood was taken from patients in case and control groups. The study was approved by Ethics Committee of the hospital. Genomic DNA was extracted from peripheral blood of all patients and control groups, using QIMamp DNA mini kit (Qiagen, US), according to the manufacturer’s instruction.

HLA- B*1502 genotype was detected by real-time PCR (RT-PCR). For this purpose, PG 1502 DNA detection kit (Pharmigene Inc., Taipei City, Taiwan) was used.

Amplification and detection was done according to the manufacturer’s instruction for applied Biosystems stepone plus.

## Results

Fifteen patients (11 males and 4 females) with severe skin drug reactions due to anticonvulsant therapy were included in this study. All of the patients were under treatment with aromatic cyclic anticonvulsants (Phenobarbital, lamotrigine, carbamazepine, and primidone) and had shown one of the severe skin drug reactions. The mean and standard deviation of age at presentation were 4.24±2.52 years. The youngest patient was 8 months old and the oldest was 8 years old. The average time interval between drug consumption and drug reaction was 14.87±5.85 days. Eight patients had an abnormal EEG; one was mental retard; seven patients had respiratory or gastrointestinal allergies or positive family history of allergy.

**Table 1:** The Number of Different Reactions Following Consumption of Each Drug

	**Phenobarbital**	**Carbamazepine**	**Lamotrigine**	**Primidone**
**SJS** *	3	2	1	0
**TEN** **	3	2	0	0
**DRESS** **	2	0	0	1
**Overlap syndrome**	0	1	0	0

* Stevens–Johnson syndrome,

** toxic epidermal necrolysis,

*** drug reaction with eosinophilia and systemic symptoms

Skin drug reactions were seen in 8 patients following phenobarbital consumption, in 5 patients for carbamazepine, in 1 patient for primidone, and 1 patient for lamotrigine. Skin drug reactions were seen in 6 patients as Stevens-Johnson syndrome, in 5 patients as toxic epidermal necrolysis, in one case as overlap syndrome and in 3 patients as DRESS. The number of different reactions following consumption of each drug is summarized in Table 1.

At the time of skin involvement, 10 patients were febrile and 4 patients had lymphadenopathy. Four patients had simple febrile convulsion, 2 had complex febrile convulsion (recurrent seizures, twice in one patient and three times in another patient) and 2 patients had one afebrile seizure (with normal neurodevelopment). 

Anticonvulsant drugs was discontinued in these patients after skin drug reaction. Seven other patients had recurrent seizures with necessity to continue anticonvulsant drugs.

The average dose of drug consumption in patients with skin reaction was 5mg/kg for phenobarbital and 13 mg/kg for carbamazepine. Brain MRI was done in 10 patients, reported abnormal in 2 of them (Brain atrophy was reported in one patient and in another one cystic encephalopathy was detected due to hypoxic-ischemic encephalopathy at birth) 

The HLA- B*1502 allele was tested in all of the patients in case and control groups. This test was negative in all patients in case group, but two patients in control group were positive for HLA- B*1502.

## Discussion

Anticonvulsant drugs are a group of pharmacological agents used in the treatment of seizures. Some patients develop hypersensitivity reactions following administration of these drugs (1, 2). A strong relationship between anticonvulsant drugs induced skin reactions and certain HLA alleles has been reported (4, 5). 

Many studies have emphasized the association between antiepileptic drug consumption and skin reactions (8-12). In our study, all of the patients have shown severe skin drug reactions due to aromatic anticonvulsant drugs consumption and most of the reactions were reported due to phenobarbital consumption. Phenobarbital is used more than other anticonvulsant drugs in our country. 

The average dose of drug consumption in patients with skin reaction was 5 mg/kg for phenobarbital and 13 mg/ kg for carbamazepine. These doses are not considered as a high level and these reactions are idiosyncratic. 

A strong association were demonstrated between HLA-B*1502 and carbamazepine-induced SJS in Asian areas including Taiwan, Hongkong, and Thailand (11). 

The association between carbamazepine-induced side effect and HLA-B*1502 allele were reported in Han Chinese of southern China. The HLA-B*1502 allele frequency in patients with SJS/TEN was significantly higher than controls and HLA-B*1502 allele had 45% positive predictive value, 100% sensitivity, 86.25% specificity and 100% negative predictive value for prediction of skin reactions(9).

HLA-B*1502 allele was seen in all of the patients with SJS due to Carbamazepine usage, but in 9% of the control population (13). The association between HLAB*1502 and carbamazepine-induced SJS/TEN was confirmed in Han Chinese and Thai populations(14, 15), but this association was not confirmed in Japan (16). 

Studies in Caucasians patients have reported no association between carbamazepine-induced SJS and HLA-B*1502, thus HLA-B*1502 allele did not exist in all ethnicities (17).

Some studies have implicated that there is an association between the HLA B*1502 as a marker for carbamazepineinduced SJS and TEN in Han Chinese, but there is no published data to show the association of HLA-B*1502 and carbamazepine-induced SJS and TEN in non-Chinese Asians. An association was shown between HLA-B*1502 and cutaneous reactions following usage of other antiepileptic drugs like phenytoin, and lamotrigine (5, 6).

We did not find any association between HLA-B*1502 and antiepileptic drug-induced cutaneous reactions to carbamazepine, lamotrigine, phenobarbital, primidone. Because of high incidence of HLA-B*1502 in many Asian populations, FDA has recommended HLA-B*1502 testing for all Asians before starting carbamazepine treatment (18). Although FDA has recommended HLA-B*1502 testing before using carbamazepine, no association between HLA- B*1502 and severe skin drug reactions was seen in this study. HLA- B*1502 testing before anticonvulsant consumption is not helpful in the Iranian population.

**In conclusion, **no association between HLA- B*1502 and severe skin reactions due to aromatic anticonvulsant drug consumption was noticed. Besides, HLA- B*1502 testing may not be helpful in prediction of severe skin reactions in Iranian population. However, another study with more samples is required to conclude precisely. 

## Authors’ contribution

Tonekaboni Sh: Study design, sampling, revising of the manuscript

Jafari N: Study design, sampling, drafting of the manuscript

Mansouri M , Jabbehdari S: Study design, sampling 

Eftekhari R: laboratory tests

Chavoshzadeh Z: Study design, sampling,

Abdollah Gorji F: Statistical analysis

Mesdaghi M: Study design, sampling, laboratory tests, revising of the manuscript

All authors agreed to be accountable for all aspects of the work in ensuring that questions related to the accuracy or integrity of any part of the work are appropriately investigated and resolved.
